# Correction to “Characteristics, Nutritional Recommendations, and Medical Interventions of 58 Dogs With Protein‐Losing Enteropathy Presenting to a Veterinary Nutrition Service”

**DOI:** 10.1111/jvim.70264

**Published:** 2025-11-06

C. M. Margrey, A. W. Rollins, M. K. Tolbert, M. Murphy, X. Zhu, and S. M. Schmid, “Characteristics, Nutritional Recommendations, and Medical Interventions of 58 Dogs With Protein‐Losing Enteropathy Presenting to a Veterinary Nutrition Service,” *Journal of Veterinary Internal Medicine* 39 (2025): e70247, https://doi.org/10.1111/jvim.70247.

In the above‐mentioned article, Table 3, columns T2 and T3 have the following corrections.

**TABLE 3:** Concurrent treatment with antibiotics, probiotics and cobalamin supplementation at each timepoint.

	T0		T1		T2		T3	
	Initial diagnosis		VNS consultation		14–90 Day recheck		Most recent recheck	
	*n* = 58		*n* = 58		*n* = 42		*n* = 48	
	Number of dogs	%	Number of dogs	%	Number of dogs	%	Number of dogs	%
Antibiotics	20	35	17	29	14	33	13	27
Tylosin	4	7	7	12	6	14	10	21
Metronidazole	14	24	9	16	7	17	3	6
Enrofloxacin	1	2	1	2	1	2	0	0
Unknown Antibiotic	1	2	0	0	0	0	0	0
Cobalamin	11	19	41	71	19	45	19	40
Enteral	7	12	14	24	9	21	7	15
Parenteral	4	7	27	47	10	24	12	25
Probiotics	12	21	23	40	13	31	12	25
LAB mixture “de Simone” formulation^a^	2	3	8	14	6	14	4	8
*Enterococcus faecium* NCIMB 30183^b^	7	7	10	17	5	12	7	15
*Enterococcus faecium* SF68^c^	1	2	4	7	1	2	1	2
Other	2	3	1	2	1	2	0	0

*Note:* Number and percentage of dogs at each timepoint administered antibiotics, probiotics, or cobalamin supplementation.

^a^ Visbiome, ExeGi Pharma LLC.

^b^ Proviable Forte or Proviable‐DC, Nutramax Laboratories, Inc.

^c^ Fortiflora, Nestlé Purina.

Column T3, Number of Dogs, row *Enterococcus faecium* NCIMB 30183, the value is 7, not 14.6.

Column T3, %, row *Enterococcus faecium* NCIMB 30183, the value is 15 not 10.

Column T2, %, row *Enterococcus faecium* SF68^C^, the value is 2, not 2.4.

Column T2, %, row Other, the value is 2, not 2.4.

The correct table is displayed here.

The authors apologize for these errors.

We apologize for this error.

